# Spatio-temporal distribution of extended spectrum β-lactamase producing *Escherichia coli* and *Klebsiella pneumoniae* blood stream infections in Laos

**DOI:** 10.1093/jacamr/dlaf180

**Published:** 2025-10-11

**Authors:** Tamalee Roberts, Vilada Chansamouth, Sayaphet Rattanavong, Viengmon Davong, Manivanh Vongsouvath, Mayfong Mayxay, Rene Neihus, David A B Dance, Ben S Cooper, Paul N Newton

**Affiliations:** Lao-Oxford-Mahosot Hospital-Wellcome Trust Research Unit, Microbiology Laboratory, Mahosot Hospital, Mahosot Road, Vientiane, Lao People’s Democratic Republic; Centre for Tropical Medicine and Global Health, Nuffield Department of Medicine, University of Oxford, Oxford, UK; Lao-Oxford-Mahosot Hospital-Wellcome Trust Research Unit, Microbiology Laboratory, Mahosot Hospital, Mahosot Road, Vientiane, Lao People’s Democratic Republic; Centre for Tropical Medicine and Global Health, Nuffield Department of Medicine, University of Oxford, Oxford, UK; Lao-Oxford-Mahosot Hospital-Wellcome Trust Research Unit, Microbiology Laboratory, Mahosot Hospital, Mahosot Road, Vientiane, Lao People’s Democratic Republic; Lao-Oxford-Mahosot Hospital-Wellcome Trust Research Unit, Microbiology Laboratory, Mahosot Hospital, Mahosot Road, Vientiane, Lao People’s Democratic Republic; Lao-Oxford-Mahosot Hospital-Wellcome Trust Research Unit, Microbiology Laboratory, Mahosot Hospital, Mahosot Road, Vientiane, Lao People’s Democratic Republic; Lao-Oxford-Mahosot Hospital-Wellcome Trust Research Unit, Microbiology Laboratory, Mahosot Hospital, Mahosot Road, Vientiane, Lao People’s Democratic Republic; Centre for Tropical Medicine and Global Health, Nuffield Department of Medicine, University of Oxford, Oxford, UK; Institute of Research and Education Development, University of Health Sciences, Vientiane, Lao People’s Democratic Republic; Saw Swee Hock School of Public Health, National University of Singapore, Singapore, Singapore; Department of Modelling, European Centre for Disease Prevention and Control (ECDC), Solna, Sweden; Lao-Oxford-Mahosot Hospital-Wellcome Trust Research Unit, Microbiology Laboratory, Mahosot Hospital, Mahosot Road, Vientiane, Lao People’s Democratic Republic; Centre for Tropical Medicine and Global Health, Nuffield Department of Medicine, University of Oxford, Oxford, UK; Faculty of Infectious and Tropical Diseases, London School of Hygiene and Tropical Medicine, London, UK; Mahidol-Oxford Tropical Medicine Research Unit, Faculty of Tropical Medicine, Mahidol University, Bangkok, Thailand; Centre for Tropical Medicine and Global Health, Nuffield Department of Medicine, University of Oxford, Oxford, UK; Mahidol-Oxford Tropical Medicine Research Unit, Faculty of Tropical Medicine, Mahidol University, Bangkok, Thailand; Lao-Oxford-Mahosot Hospital-Wellcome Trust Research Unit, Microbiology Laboratory, Mahosot Hospital, Mahosot Road, Vientiane, Lao People’s Democratic Republic; Centre for Tropical Medicine and Global Health, Nuffield Department of Medicine, University of Oxford, Oxford, UK; Mahidol-Oxford Tropical Medicine Research Unit, Faculty of Tropical Medicine, Mahidol University, Bangkok, Thailand

## Abstract

**Objectives:**

ESBLs are an important cause of third generation cephalosporin resistance in Enterobacterales. However, there is a paucity of data on ESBLs in blood stream infections (BSI) in Laos. The aim of this study was to investigate the presence of ESBL-producing *Escherichia coli* (ESBLEC) and ESBL-producing *Klebsiella pneumoniae* (ESBLKP) in blood cultures submitted to Mahosot Hospital, Laos and how these have changed over 18 years.

**Methods and materials:**

This retrospective observational study included blood cultures from patients presenting with fever to Mahosot Hospital between 2000 and 2018. Full identification and antibiotic susceptibility testing was carried out on positive bottles. ESBL production was determined using the double-disc method. Patient clinical and residence data were included in univariable and multivariable analyses to identify risk factors for having an ESBL.

**Results:**

From 52 249 blood culture sets collected over the 18-year period, 222 (0.42%) were positive for an ESBLEC or ESBLKP. The proportion of *E. coli* and *K. pneumoniae* isolates that were ESBL positive increased from 3.2% in 2000 to 35.4% in 2018. While there was a steady increase seen in ESBLEC over the 18-year period, ESBLKP have remained at ∼7% of *K. pneumoniae* isolates. Most ESBLs were community acquired. From univariable analysis, factors positively associated with an ESBL isolate included chronic renal failure, renal stones and having taken an antibiotic in the week before the blood sample.

**Conclusion:**

With a rising trend of ESBLEC in Laos, controlling unregulated antibiotic usage in the community will be pivotal to stopping further increases.

## Introduction

Blood stream infections (BSI) and sepsis are important causes of morbidity and mortality worldwide with mortality rates as high as 40% in developing countries.^1^ There is limited information on the causes of BSI in low- and middle-income countries, but this information is crucial for guiding antibiotic therapy, clinical management and preventative measures. The rise in the prevalence of bacteria resistant to commonly used antibiotics has increased the importance of surveillance for BSI globally. ESBL enzymes have the ability to hydrolyse third generation cephalosporins (3GC) and are the most common mechanism of cephalosporin drug resistance in Enterobacterales, with the prevalence increasing around the world.^2^ In the Greater Mekong Sub-region, the proportion of ESBL-producing *E. coli* (ESBLEC) in clinical blood samples increased by 13.2% per year over two decades (2000–2020). However, there was no significant trend seen for ESBL-producing *K. pneumoniae* (ESBLKP) during the same time period.^3^

Lao People’s Democratic Republic (Laos) is a land-linked country in Southeast Asia with a population of 7 664 993 people in 2023.^4^ There is a seasonal climate, with dry cool months from November to February, dry hot months from March to May, and a wet season from late May to October. Although ESBL-producing Enterobacterales are a major cause of morbidity and mortality in Southeast Asia, epidemiological understanding of ESBLs in Laos is limited. One study found an increase from 7.8% to 34.7% of ESBLEC over a 5-year period in Laos^5^ while a study on Lao pre-school children found 23% colonized with ESBL-producing bacteria.^6^ Two molecular studies on ESBLs in Laos found that all ESBLEC harboured *bla*_CTX-M_ which is similar to other molecular studies from Asia^6,7^ while ESBLKP were found to harbour *bla*_SHV-2A_ and *bla*_CTX-M_ variants.^6^

The spatial distribution of ESBLEC and ESBLKP in Laos have not been described and it is not known whether they have similar distribution and prevalence across the country and whether antimicrobial susceptibility patterns have changed over time. We therefore describe the spatio-temporal epidemiology of patients with ESBL-positive *E. coli* and *K. pneumoniae* BSI admitted to Mahosot Hospital, Vientiane, Laos over an 18-year period.

## Methods and materials

### Study samples

All blood culture isolates identified by the Microbiology Laboratory at Mahosot Hospital (17°57′36.2′′N 102°36′43.3′′E) from patients presenting with fever and admitted to Mahosot Hospital, Vientiane Capital, between February 2000 and December 2018 were included in the study. Mahosot Hospital is a ∼400-bed government hospital providing primary, secondary and tertiary care and admitting ∼2000 patients/month.^8^ Blood culture bottles were incubated in air at 37°C for 7 days (see Phetsouvanh *et al.*^9^). Bottles were examined daily for turbidity and were sub-cultured onto blood and chocolate agar if positive. ‘Blind’ subcultures were also performed on days 1 and 7 post-inoculation. Bacteria were identified using standard microbiological techniques and API 20E (bioMerieux, Marcy L’Etoile, France). Comprehensive antimicrobial susceptibility testing (AST) profiles were determined using disc diffusion on Mueller-Hinton agar (Oxoid) following CLSI guidelines for the year the sample was collected and were all subsequently re-interpreted using 2018 CLSI guidelines (*M100*, Twenty-Eighth edition, January 2018). ESBL production was determined for *E. coli* and *K. pneumoniae* isolates using the double-disc method [cefotaxime±clavulanate and ceftazidime ± clavulanate (BD)] following CLSI guidelines.

### Statistical analysis

The incidence of bloodstream infections from which ESBL-producing organisms were isolated was calculated as the number of reported ESBL BSIs per 10 000 or 100 000 people from each district and province using population census data from 2005 and 2015.^10,11^ Owing to uncertainty about health-seeking behaviour and access, and in the absence of data on community BSI incidence, reported incidence rates will represent a lower bound for the true disease burden. Data were analysed using Stata version 14 (StataCorp, College Station, TX, USA). The percentage of samples that were taken ≤2 days after admission for ESBL-positive versus EBSL-negative *E. coli* and *K. pneumoniae* isolates were compared using the Pearson chi-squared test. Univariable and multivariable logistic regression models were used to determine factors associated with infection with an ESBL-positive organism. Variables were selected based on biological plausibility and substantive knowledge (variables shown in Tables 1 and 2). For patients who did not grow an *E. coli* or *K. pneumoniae*, the first blood culture was included for the univariable and multivariable logistic regression. The two-sample Wilcoxon rank-sum (Mann–Whitney) test was used to compare median age of patients and median distance from patient village to Mahosot Hospital for the different ESBL versus non-ESBL groups. Mapping of cases was performed using QGIS version 2.14.2.

**Table 1.** Univariable and multivariable logistic regression of factors associated with an ESBL in ESBL-positive and -negative *E. coli* and *K. pneumoniae*

Factor	No. patients with answer	No. patients with factor	No. ESBL-positive patients with factor	Univariable analysis			Multivariable analysis		
				OR	95% CI	*P* value	OR	95% CI	*P* value
Gender (male)	1142	451 male, 691 female	90 male, 131 female	0.94	0.70–1.27	0.677	1.30	0.66–2.54	0.451
Fever	1122	1083	210	1.57	0.35–6.99	0.556	1.00	Omitted	
Rigours	1113	730	128	0.72	0.53–0.98	0.038	0.48	0.24–0.98	0.043
Headache	1110	615	104	0.71	0.53–0.96	0.023	0.77	0.38–1.56	0.471
Arthralgia	1104	359	104	0.87	0.63–1.20	0.392	0.64	0.29–1.45	0.288
Jaundice	1108	231	52	1.29	0.91–1.84	0.155	2.44	1.18–5.05	0.017
Vomiting	1109	319	65	1.09	0.79–1.51	0.596	1.10	0.54–2.24	0.789
Dysuria	1089	195	35	0.88	0.59–1.32	0.555	0.60	0.23–1.59	0.307
Diarrhoea	1110	229	31	0.59	0.40–0.90	0.014	0.94	0.42–2.12	0.882
Abdominal pain	1101	351	62	0.87	0.62–1.2	0.387	1.01	0.51–2.02	0.973
Drowsy	1101	182	38	1.13	0.76–1.67	0.543	0.96	0.40–2.28	0.922
Diabetes	846	300	60	1.27	0.88–1.82	0.201	1.02	0.49–2.08	0.967
Chronic renal failure	719	64	21	2.68	1.53–4.70	0.001	5.22	1.63–16.63	0.005
Renal stones	685	73	32	4.65	2.78–7.77	0.000	2.69	0.95–7.63	0.062
Antibiotic last week	610	162	63	3.82	2.53–5.77	0.000	3.84	2.04–7.22	0.000

**Table 2.** Univariable and multivariable logistic regression of factors associated with an ESBL from all patients who had a blood culture taken

Factor	No. patients with answer	No. patients with factor	No. patients ESBL positive with factor	Univariable analysis			Multivariable analysis		
				OR	95% CI	*P* value	OR	95% CI	*P* value
Gender (male)	45 050	24 618 male, 20 432 female	90 male, 131 female	1.82	1.39–2.39	0.000	2.16	1.28–3.65	0.004
Fever	44 700	39 774	210	3.69	1.74–7.84	0.001			
Rigours	44 277	16 131	128	2.59	1.97–3.41	0.000	2.49	1.46–4.27	0.001
Headache	44 426	23 952	104	0.79	0.60–1.04	0.090	0.62	0.36–1.09	0.095
Arthralgia	44 104	11 411	104	1.19	0.89–1.61	0.244	0.39	0.19–0.78	0.008
Jaundice	44 127	5739	52	2.09	1.52–2.87	0.000	3.19	1.83–5.54	0.000
Vomiting	44 397	11 960	65	1.17	0.87–1.57	0.286	1.24	0.70–2.18	0.461
Dysuria	43 814	2563	35	3.27	2.27–4.71	0.000	1.15	0.49–2.72	0.742
Diarrhoea	44 323	9300	31	0.64	0.44–0.94	0.024	0.76	0.38–1.52	0.430
Abdominal pain	43 947	9097	62	1.55	1.14–2.09	0.004	1.50	0.84–2.66	0.172
Drowsy	43 937	5358	38	1.59	1.12–2.27	0.009	1.42	0.72–2.81	0.305
Diabetes	28 987	3734	60	4.62	3.32–6.42	0.000	2.43	1.28–4.63	0.007
Chronic renal failure	27 190	828	21	6.83	4.25–11.00	0.000	2.44	1.06–5.61	0.036
Renal stones	25 807	854	32	11.25	7.46–16.98	0.000	4.48	2.10–9.56	0.000
Antibiotic last week	22 510	7382	63	1.99	1.40–2.83	0.000	2.08	1.26–3.44	0.004

### Quality control

For identification and AST, internal quality control was completed for all media and antibiotics following the laboratory’s internal quality control routine protocols. The Microbiology Laboratory has been participating in the UK National EQ Scheme since 2006. Results are reported following the MICRO Checklist (File S1, available as Supplementary data at *JAC-AMR* Online).^12^

### Ethics

Ethics approval for the blood culture study was obtained from the Oxford Tropical Research Ethics Committee (UI study ethics approval 41-20) and from the Lao National Ethics Committee for Health Research (716/REC).

## Results

There were 52 249 blood culture sets collected from 45 163 patients during the 18-year period; 222/45 163 patients (0.49%) were positive for an ESBLEC or ESBLKP. There were 177 (21%) ESBLEC from a total of 834 *E. coli* and 45 (14.5%) ESBLKP from a total of 310 *K. pneumoniae* isolates. Sixty-seven *E. coli* or *K. pneumoniae* positive patients had more than one blood culture taken. Patients with duplicate samples were not more likely to have an ESBL, with only 12/67 of such patients having an ESBL-positive isolate. Of all patients who had more than one blood culture set taken and grew an *E. coli* or *K. pneumoniae*, there was only one patient in whom the initial sample was ESBL negative and the second sample ESBL positive, with the ESBL-positive sample taken a year later. All patients with duplicate samples that had an initial ESBL-positive isolate remained positive for all subsequent samples. Blood cultures from five patients grew both *E. coli* and *K. pneumoniae*, neither of which were ESBL-positive. Therefore, 61/67 patients with an *E. coli* or *K. pneumoniae* had duplicate isolates removed from the dataset for analysis, with the initial sample included.

**Figure 1.**
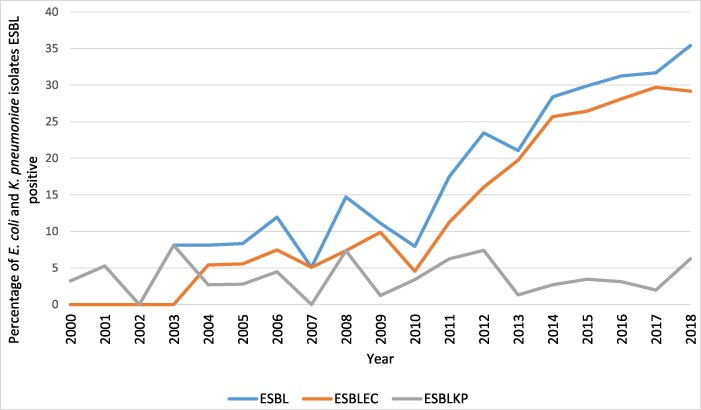
Percentage of all *E. coli* and *K. pneumoniae* blood culture isolates that were ESBL positive per year, the percentage of *E. coli* that were ESBL positive (ESBLEC) and the percentage of *K. pneumoniae* that were ESBL positive (ESBLKP).

The first reported ESBL was an ESBLKP in 2000 and the first ESBLEC was reported in 2004. There has been a continuous increase in the percentage of *E. coli* and *K. pneumoniae* that were ESBL positive, from 3.2% (1/31) ESBL-positive in 2000 to 35.4% (34/96) positive in 2018 (Figure 1). While there was a steady increase seen in ESBLEC over the 18-year period, ESBLKP have remained at ∼7% of all *K. pneumoniae* isolates.

### Antibiotic susceptibility test results

**Figure 2.**
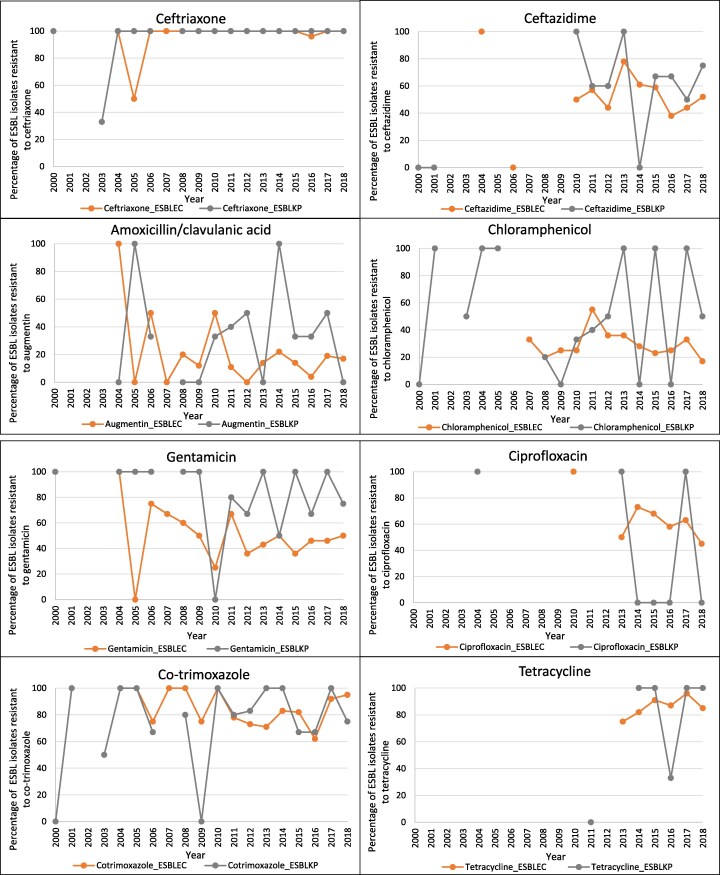
Proportion of ESBL-positive *E. coli* and *K. pneumoniae* isolates resistant to individual antibiotics over time.

Antibiotic susceptibility test results for *E. coli*, ESBLEC, *K. pneumoniae* and ESBLKP are shown in Table S1. All ESBL-positive isolates that were tested against a carbapenem were susceptible (*n* = 224). There was only one ESBLEC that was amikacin resistant; all ESBLKP isolates were amikacin susceptible. ESBLEC and ESBLKP isolates differed in frequency of resistance to amoxicillin/clavulanic acid (15.8% versus 32.5%), ciprofloxacin (60.0% versus 26.7%), ofloxacin (66.2% versus 16%), gentamicin (47.2% versus 77.5%) and chloramphenicol (28.8% versus 46.3%) but there were no substantial differences in resistance to ceftazidime (52.7% versus 57.1%), amikacin (0.8% versus 0%), co-trimoxazole (82.1% versus 77.3%) or tetracycline (87.83% versus 76.9%). There were also notable differences in the number of resistant isolates for the above antibiotics between both the non-ESBLEC and ESBLEC groups and between the non-ESBLKP and ESBLKP groups, with the exception of chloramphenicol for non-ESBLEC versus ESBLEC and tetracycline for both non-ESBLEC versus ESBLEC and non-ESBLKP versus ESBLKP. Figure 2 shows the yearly trends of resistance for ESBLEC and ESBLKP isolates, with a decrease in the percentage of ESBLEC isolates resistant to amoxicillin/clavulanic acid and chloramphenicol, both dropping below 40% resistant since 2012. Co-trimoxazole resistance frequency has stayed above 60% since 2010 for both ESBLEC and ESBLKP and gentamicin resistance frequency has remained around 40% or above since 2011, with a higher resistance rate seen in ESBLKP then ESBLEC.

### Demographic results

For all ESBL-positive patients the median age was 53 years (range 2 days–93 years) with the highest frequency in the 56–60 year age group (Figure S1). The median age for ESBLKP was substantially lower than ESBLEC: 22 years (range 2 days–85 years) compared with 56 years (range 13 days–93 years), respectively, but was similar for susceptible *K. pneumoniae* and *E. coli*, which had respective median ages of 55 years (range 0 days–98 years) and 58 years (range 0 days–98 years). There were 131 (59.3%) females and 90 (40.7%) males who were ESBL positive. The data from all patients were included in the study although denominators vary for different parameters due to incomplete data. While fever was the key indication for taking a blood culture, not all patients had fever recorded on the form that was completed at the time the blood cultures were collected. All ESBL-positive patients had fever, 128 (59.5%) from 215 with responses had rigours and 104 (48.6%) from 214 responses had headache. Sixty-three (49.6%) patients from 127 responses reported having taken an antibiotic in the week before admission. Patient outcome data were not available for most ESBL-positive patients (205/222) due to self-discharge and lack of a formal follow-up system but 10 patients were known to have died in hospital. For ESBL-positive patients, 220/222 (99%) had both specimen and admission dates with the median interval between date of admission and sample date being 1 day (range 0–30 days). For ESBL-positive patients 156/220 (70.1%) had a specimen date ≤2 days since admission. ESBL-negative *E. coli* and *K. pneumoniae* patients had a median of 0 days from admission to a sample being taken (range 0–41 days), with 772/911 (84.7%) having a specimen date ≤2 days since admission. There were only 16 ESBL-positive patients and 116 ESBL-negative patients who had a recorded discharge date so further comparisons were not possible. From the univariable logistic regression using all *E. coli* and *K. pneumoniae* isolates, there were six factors associated with an ESBL-positive specimen. Factors positively associated with an ESBL isolate in the univariable analysis included chronic renal failure, renal stones and having taken an antibiotic in the last week (Table 1). Factors negatively associated with an ESBL included rigours, headache and diarrhoea. From the multivariable logistic regression, factors positively associated with a patient having an ESBL were jaundice, chronic renal failure and having taken an antibiotic in the last week. For univariable logistic regression for all patients who had a blood culture taken, factors positively associated with an ESBL-positive organism included male gender, fever, rigours, jaundice, dysuria, abdominal pain, drowsiness, chronic renal failure, renal stones and having taken an antibiotic in the last week (Table 2). Factors positively associated with an ESBL-positive organism from the multivariable logistic regression for all patients who had a blood culture taken included male gender, rigours, jaundice, diabetes, chronic renal failure, renal stones and having taken an antibiotic in the last week. May and August saw the highest percentage of ESBLEC isolates, while the highest percentage of ESBLKP isolates were seen in March and November (Figure S2).

### Spatial results

**Figure 3.**
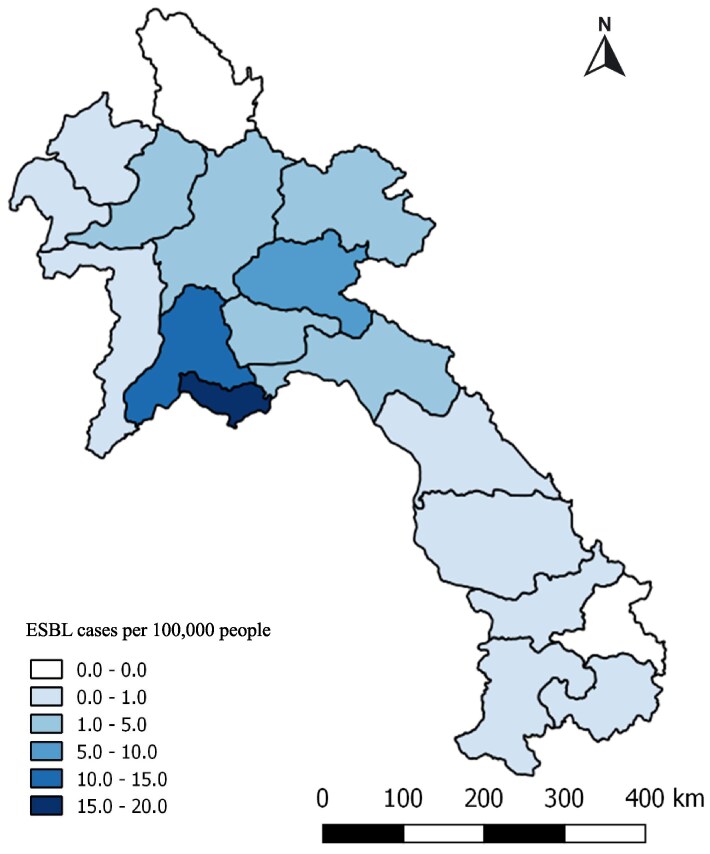
Total combined ESBLEC and ESBLKP cases per 100 000 people per province from 2000 to 2018.

District and province locations were available for the homes of all ESBLEC patients and 43/45 ESBLKP patients. Most ESBL-positive patients came from Vientiane Capital, the main catchment area for Mahosot Hospital (136/220 patients, 61.8%, 17.45/100 000 people), followed by adjacent Vientiane Province (39/220 patients, 17.7%, 9.59/100 000 people) (Figure 3). ESBL-positive patients came from 16 of the 18 Lao Provinces with no patients from Phongsaly and Sekong Provinces (the number of *E. coli* and *K. pneumoniae* isolates by province and the proportion that were ESBL positive are presented in Table S2). At a district level, Sikhottabong district in Vientiane Capital had the highest number of ESBL-positive patients (39/220 patients, 17.8%, 3.22/10 000 people) followed by Chantabuly district, also in Vientiane Capital (21/220 patients, 9.6%, 3.04/10 000 people) (Figure S3).

Village location information was available for 210/222 (94.6%) ESBL-positive patients and 735/922 (79.7%) non-ESBL *E. coli* and *K. pneumoniae* patients. The median distances for all ESBL-positive patients, ESBLEC and ESBLKP patients from Mahosot Hospital were all similar: 14.78 km (range 0.6–519 km), 15.03 (range 0.6–519 km) and 17.17 (range 1.8–398 km), respectively. This is compared with the median distance of home village to Mahosot Hospital for non-ESBL *E. coli* and *K. pneumoniae* patients that was closer at 8 km (range 0.34–457 km).

## Discussion

This study is the largest epidemiological description of ESBL-positive BSI patients in Laos to date. Overall, 21% of *E. coli* isolates were ESBL-positive and 14.5% of *K. pneumoniae* isolates were ESBL-positive. There has been a steady increase in the proportion of *E. coli* isolates that are ESBL-positive over the 18 years, with ∼35% of *E. coli* isolates ESBL-positive in 2018. This increase could be due to several factors including intrinsic dynamics, the uncontrolled use of antibiotics in health facilities, the community and livestock industry, spread of ESBLs from neighbouring countries and also an increase in access to healthcare over the time period.^13^ While this study was based on data from inpatients at Mahosot Hospital in the capital city of Vientiane, and therefore is not a true reflection of the spatial distribution of ESBLs in the country, during the study period Mahosot Hospital Microbiology Laboratory was the main microbiology laboratory in the country and the only one undertaking a significant amount of bacterial culture and susceptibility testing on diagnostic clinical samples.

There were differences in the frequency of resistance seen for all antibiotics except tetracycline and chloramphenicol for the non-ESBLEC group versus the ESBLEC group, and for all antibiotics except tetracycline for the non-ESBLKP versus ESBLKP group. Significant differences in susceptibility patterns were also seen from a study in adjacent Thailand between the ESBL-negative and -positive groups for amoxicillin, ceftazidime, cefotaxime, gentamicin, ciprofloxacin and co-trimoxazole.^14^ In Australia, ESBLs were least resistant to gentamicin (21.8% resistant) for all the antibiotics analysed,^15^ which is not the case in Laos where there were differences between the proportion of resistance for the non-ESBL groups versus the ESBL groups for gentamicin, with ESBLKP having the highest overall gentamicin resistance at 77.5%. Ceftazidime, an antibiotic used to screen for ESBL production in the laboratory, has shown resistance levels below 80% in ESBLs since 2014. That ceftriaxone, another screening antibiotic, had resistance levels of 100% for all ESBL isolates except one since 2006, suggests that in this setting, ceftriaxone resistance is a much better screening tool.

There were no treatment guidelines for ESBL BSI in Laos during the study period, with treatment predominantly based on individual patient antibiotic susceptibility testing results. If an infection is caused by an organism resistant to first line antibiotics, then meropenem may be advised but as this drug is expensive in Laos, and was not readily accessible during the study period, amikacin was commonly used, however, data on usage for both of these antibiotics are not available for the study period. The monitoring of serum aminoglycoside levels is also not available in the country. The first guidelines were developed in 2021 but there are no guidelines for EBSL BSI; for upper urinary tract infections meropenem or a combination of drugs, including ceftazidime, amikacin and metronidazole or ciprofloxacin and metronidazole, are suggested. Country-wide AMR surveillance and monitoring, including carbapenem resistance, started in 2020 partially supported by the Fleming Fund, and antimicrobial usage point prevalence studies have also been carried out in the country since 2017.^16,17^ Continual surveillance will be important to see whether resistance rates increase.

There was a difference in the proportion of ESBL-positive patients compared with ESBL-negative patients who had a blood culture taken ≤2 days after admission, and with most ESBL-positive patients (70.1%) having had samples taken ≤ 2 days after admission it can be assumed that in these patients the BSI was community acquired. A study in northern Vietnam estimated similar proportions of ESBLEC in hospital- and community-acquired BSI but found a higher proportion of ESBLKP BSI cases that were hospital acquired compared with community acquired.^18^ Another study from Vietnam found most ESBL BSIs to be community acquired and demonstrated an increase from 45% to >60% of *E. coli* BSI that were ESBL in the community over a 4-year period.^19^ A study from northern Thailand found 11.8% and 11.4% of community-acquired *E. coli* and *K. pneumoniae* BSIs, respectively, were ESBL-positive and there was an increase in community-acquired ESBL BSI seen over time.^20^ Another study in two rural provinces in Thailand found an increase in the frequency of community-acquired BSI (defined as a positive blood culture taken in the first 72 h of hospital admission) ESBLEC over a 7-year period.^14^ However, the study also found that there was an increase in the number of community-acquired ESBL BSIs, hospital-acquired BSIs were twice as likely to be ESBL. The percentage of ESBLEC BSI ranged from 23.8% to 31.9% by age group.

ESBL BSI are probably more likely to be community acquired than hospital acquired in Laos due to the easy access to antibiotics in the community without a prescription, close proximity to livestock in the household and closely knit communities where ESBLs could easily be spread. Patients in Laos also do not usually stay long in hospital as cost and care are borne by the patient and their family so there is limited time for people to acquire an ESBL in hospital. The spread of ESBLs from colonized people in the community due to faecal carriage has also been shown, with a study on ESBLs in children from childcare centres in Vientiane Capital and Vientiane Province finding a colonization prevalence of 23.2% from 397 individuals sampled.^6^

Previous studies have found risk factors associated with ESBL bacteraemia included previous urinary tract infections, nasogastric tube catheterization, prior antibiotic exposure (especially cephalosporins) and longer duration of hospitalization.^21–23^ Our results are similar to these with risk factors positively associated with an ESBL being chronic renal failure, jaundice and antibiotics taken in the last week. There was a much younger age seen for ESBLKP patients compared with ESBLEC and ESBL-negative *K. pneumoniae* patients.

ESBL-positive patients were more likely to come from further away from Mahosot Hospital than EBSL-negative patients. Why is unclear but could be due to self-treatment for infection and delayed presentation to hospital when infection becomes severe due to the distance needed to travel and lack of local healthcare services. This was also seen in Australia with increased ESBLEC incidence in rural areas and it was suggested that this could be related to impaired access to healthcare services.^24^

There are several limitations to this study. The data presented here cover an 18-year period to the end of 2018. In 2019, the laboratory changed to an automated blood culture system and changed antibiotic susceptibility testing from CLSI to the EUCAST. In 2020 inter-province travel was restricted due to the SARS CoV-2 pandemic, which was only lifted in March 2022. In November 2021 the laboratory and several wards moved to a newly built building which disrupted hospital admissions for several months. Because of these reasons, we did not include more recent data. There could also be reporting bias in the univariable and multivariable analysis leading to a positive association for specific clinical condition risk factors. It was also not possible to study the resistance genes present and their location (whether chromosome or plasmid), which could have implications for transmission. Further studies to include the data from recently introduced surveillance sites as well as sequencing data would add to the current knowledge of the epidemiology of ESBLs in Laos and inform interventions to reduce their frequency and impact. Although this is the first large scale epidemiological report on ESBL BSI in Laos, it could potentially be either an underestimation or overestimation of the true incidence due to only including hospitalized patient data from a single site. Further information on length of stay and discharge status would help determine clinical outcomes for patients with a BSI caused by an ESBL.

### Conclusions

The rising trend of ESBL-positive *E. coli* in Laos is cause for concern. The use of antibiotics in the community and in animals, access to clean water and sanitation, and the spread of ESBLs in the environment are areas for further investigation and control to stop further increasing trends.

## Supplementary Material

dlaf180_Supplementary_Data

## Data Availability

All data are included in the submitted text and tables. Data are available on request from the MORU Data Access Committee (datasharing@tropmedres.ac).

## Supplementary data

File S1, Figures S1 to S3 and Tables S1 and S2 are available as Supplementary data at *JAC-AMR* Online.
